# Transcriptome analysis reveals long intergenic non-coding RNAs involved in skeletal muscle growth and development in pig

**DOI:** 10.1038/s41598-017-07998-9

**Published:** 2017-08-18

**Authors:** Cheng Zou, Jingxuan Li, Wenzhe Luo, Long Li, An Hu, Yuhua Fu, Ye Hou, Changchun Li

**Affiliations:** Key Lab of Agriculture Animal Genetics, Breeding, and Reproduction of Ministry of Education, College of Animal Sciences and Technology, Huazhong Agricultural University, Wuhan, 430070 People’s Republic of China

## Abstract

Long intergenic non-coding RNAs (lincRNAs) play essential roles in numerous biological processes and are widely studied. The skeletal muscle is an important tissue that plays an essential role in individual movement ability. However, lincRNAs in pig skeletal muscles are largely undiscovered and their biological functions remain elusive. In this study, we assembled transcriptomes using RNA-seq data published in previous studies of our laboratory group and identified 323 lincRNAs in porcine leg muscle. We found that these lincRNAs have shorter transcript length, fewer exons and lower expression level than protein-coding genes. Gene ontology and pathway analyses indicated that many potential target genes (PTGs) of lincRNAs were involved in skeletal-muscle-related processes, such as muscle contraction and muscle system process. Combined our previous studies, we found a potential regulatory mechanism in which the promoter methylation of lincRNAs can negatively regulate lincRNA expression and then positively regulate PTG expression, which can finally result in abnormal phenotypes of cloned piglets through a certain unknown pathway. This work detailed a number of lincRNAs and their target genes involved in skeletal muscle growth and development and can facilitate future studies on their roles in skeletal muscle growth and development.

## Introduction

In mammals, the majority of transcribed loci have very weak or no protein-coding potential, and many of these loci are long (>200 nucleotides in length) intergenic RNAs^1, 2^. Although lincRNAs have been regarded as transcription noise for a long time because of its lower expression and protein-coding potential compared with protein-coding genes^3^, increasing evidence indicate that lincRNAs have various roles in many biological processes such as reprogramming^4^, embryonic development^5, 6^, and skeletal muscle development^7, 8^.

In the previous studies of our laboratory group, we reported some methylome and transcriptome differences between abnormal cloned piglets (the abnormal cloned group; ab), normal cloned piglets (the normal cloned group; nc), and conventionally bred piglets (the normal *in vivo* group; nv) and found some skeletal-muscle-related mechanisms that may contribute to the cloned piglets’ abnormalities such as standing, walking and eating disabilities, in the perspective of gene expression and methylation^9, 10^. Based on previous studies, we wanted to explore whether or not lincRNAs were one of the causes of the abnormalities in the cloned piglets.

In recent decades, with the development of RNA sequencing technology, several lincRNAs in different species and tissues have been identified and characterized^11, 12^. In pigs, Zhou and Zhao have identified 6,621 and 570 lincRNA transcripts, and their work largely enriched pig lincRNA annotation^7, 13^. A series of lincRNAs such as *linc-YY1*, *lincRNA-p21*and *linc-MYH* have been proven to have an impact on muscle growth^14–16^. Although many lincRNAs have been identified in pig, there are still lots of lincRNAs remaining undiscovered compared with human and mouse^17, 18^. The relationship between DNA methylation and lincRNA expression in pig has not been reported, and lincRNAs that were involved in skeletal muscle growth and development of pig are yet to be elucidated.

In this study, we used differential expression analysis to explore the lincRNAs that may contribute to the abnormalities of cloned piglets. We carried out transcriptome assembly of leg muscle transcriptomes of three groups which were studied in our previous research^9, 10^. We identified a total of 323 putative lincRNAs and characterized the basic feature of these lincRNAs. We then profiled the expression of those lincRNAs in all groups and detected some differentially expressed lincRNAs. Association analysis of DNA methylation and expression of lincRNA genes revealed that methylation in the promoter of lincRNA genes can slightly down-regulated its expression. Gene ontology and pathway analysis was performed on potential target gene (PTGs) of identified lincRNAs. Moreover, the relationship between the methylation and expression of lincRNAs and the expression of PTGs were determined successfully. This study provides novel insights into the research on factors that affect pig muscle growth and development.

## Results

### Transcriptome reconstruction and lincRNA identification

**Figure 1:**
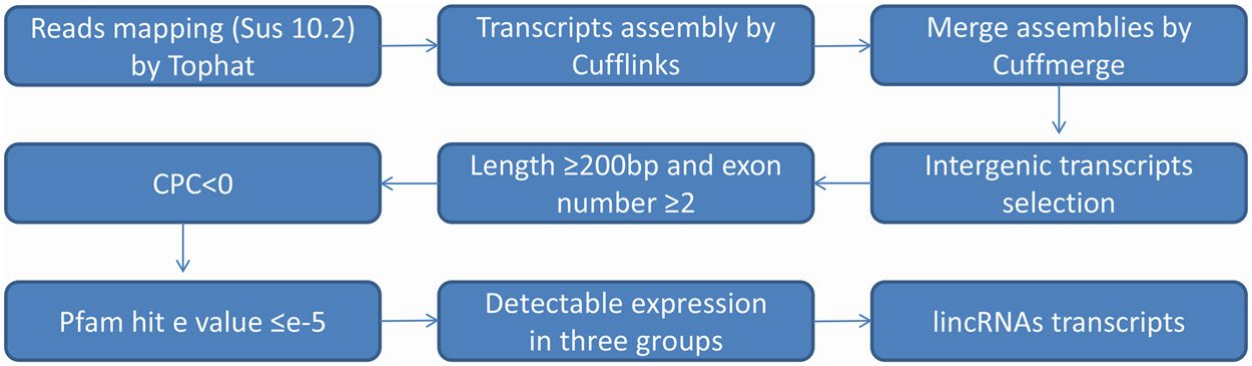
Overview of the identification pipeline for lincRNAs in this study. See the main content for details.

To identify and analyze the lincRNAs involved in fetal porcine skeletal muscle growth and development, we used RNA-seq data from our previous study that included three study groups^9, 10^. We identified lincRNAs based on the illustration shown in Fig. 1.

**Figure 2:**
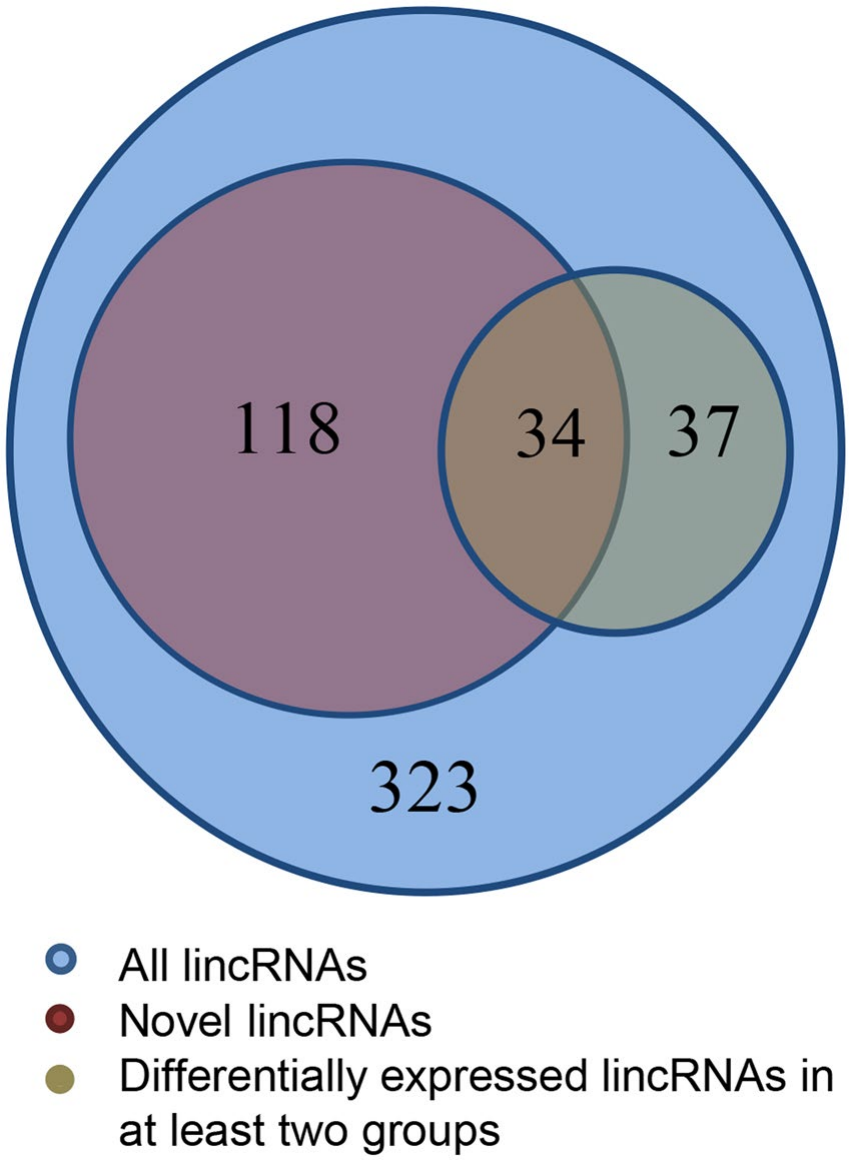
Statistics of different kinds of lincRNAs. The blue circle indicates all lincRNAs identified in this study, the red circle indicates the novel lincRNAs and the brown circle indicates the lincRNAs that differentially expressed in at least two groups.

Each RNA-seq dataset was first mapped to the whole genome of *Sus scrofa*a (10.2) separately and 40 million mapped reads were retained (Table 1). Then, we reconstructed transcriptome for each group through Cufflinks^19^. All transcripts were pooled and merged into a nonredundant transcript set through Cuffmerge. We obtained a total of 47,130 transcripts and 11,407 of which were intergenic transcripts. After filtering transcripts by length and exon number, 780 transcripts remained. We then evaluated the coding potential of remaining transcripts with the Coding Potential Calculator (CPC)^20^. All transcripts with CPC scores >0 were discarded and 501 transcripts were retained. To guarantee the thorough elimination of protein-coding transcripts, transcripts that encoded any of the known protein domains catalogued in the Pfam protein family database were filtered out and 345 transcripts were reserved. To obtain high confidence transcripts, we stipulated transcripts must have detectable expression in all groups. Finally, we obtained 323 putative lincRNAs encoded by 306 loci which were distributed in all chromosomes except the Y chromosome, and 152 of these 323 lincRNAs have no overlap with currently annotated coding or noncoding transcripts (Fig. 2, Table S1).

**Table 1:** Mapped resds number and sample source of MeDIP-seq RNA-seq data.

	Abnormal cloned group		Normal cloned group		Normal *in vivo* group	
	MeDIP-seq	RNA-seq	MeDIP-seq	RNA-seq	MeDIP-seq	RNA-seq
Mapped reads	38,944,094	12,687,738	39,769,050	14,078,937	38,427,768	13,317,949
GEO number	GSM1246252	GSM1241829	GSM1246253	GSM1241830	GSM1715566	GSM1715563

MeDIP-seq: methylated DNA immunoprecipitation sequencing.

### Genomic characters of lincRNAs

**Figure 3:**
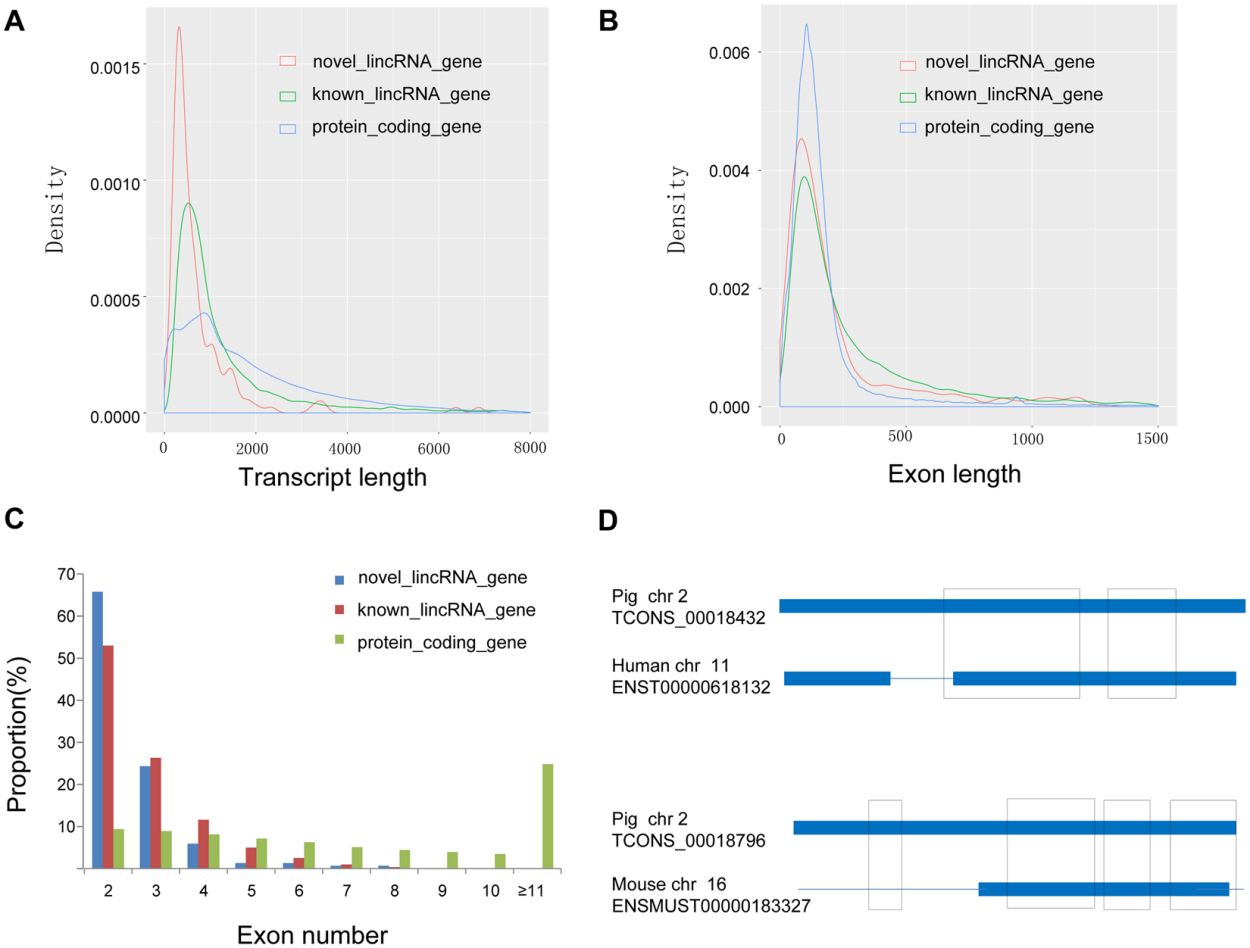
Comparisons of transcript length, exon length and exon number between novel lincRNA genes, known lincRNA genes and protein-coding genes. (**A**) Comparisons of transcript length. Novel lincRNA genes show shorter average transcripts length (776 bp) than that of the known lincRNA genes (1,361 bp) and the protein-coding genes (1,828 bp); (**B**) Comparisons of exon length. Novel lincRNA genes show shorter mean exon length (307 bp) than that of the known lincRNA genes (450 bp) but longer than that of the protein-coding genes (233 bp); (**C**) Comparisons of exon number. Novel lincRNA genes trend to have less exons than that of the known lincRNA genes (2.5 vs 2.8) and protein-coding genes (2.5 vs 7.8); (**D**) Representative images of conserved regions between pig lincRNAs with human and mouse lincRNAs. Thick lines indicate an exon and thin lines indicates an intron of the lincRNAs. Boxes indicate the conserved region between two lincRNAs.

Based on the reconstructed transcriptome, we also obtained 31,744 protein-coding transcripts that corresponded to 25,153 genes, indicating an average of 1.3 isoforms per protein gene. Moreover, 12,103 known lincRNA transcripts corresponded to 7,381lincRNA genes in the pig lincRNA annotation files. The average transcripts length of novel lincRNA genes in our study was 776 bp, which was significantly shorter than that of the known lincRNA genes and protein-coding genes (novel lincRNA genes vs known lincRNA genes: 776 bp vs 1361 bp, P < 2.2e-16, Wilcox.test; novel lincRNA genes vs protein-coding genes: 776 bp vs 1828 bp, P < 2.2e-16, Wilcox.test) (Fig. 3A). Meanwhile, the mean exon length of novel lincRNA genes was significantly shorter than that of the known lincRNA genes but longer than that of the protein-coding genes (novel lincRNA genes vs known lincRNA genes: 307 bp vs 450 bp, P < 7.8e-11, Wilcox.test; novel lincRNA genes vs protein-coding genes: 307 bp vs 233 bp, P < 1.4e-3, Wilcox.test) (Fig. 3B). Furthermore, we found that lincRNA genes trend to have less exons than known lincRNA genes and protein-coding genes (novel lincRNA genes vs known lincRNA genes: 2.5 vs 2.8, P < 3.8e-4, Wilcox.test; novel lincRNA genes vs protein-coding genes: 2.5 vs 7.8, P < 2.2e-16, Wilcox.test). The number of exon distribution showed that more than 90.1% and 79.3% of the novel and known lincRNA transcripts contained less than 4 exons, whereas only 18.3% of protein-coding transcripts contained less than 4 exons (Fig. 3C). The characteristics of novel lincRNA genes such as shorter transcripts length, longer exons length and fewer number of exons compared with protein-coding genes were in accordance with previous studies^5, 11, 13^.

To identify the sequence conservation of the pig lincRNAs in other mammals, we aligned the pig lincRNAs with those of human and mouse using BLASTN. We found 12 and 7 of 152 novel pig lincRNAs, 728 and 134 of 7,381 known pig lincRNAs had significantly alignments with human and mouse lincRNAs (E-value < 10^−5^), respectively. Besides, we also found that majority of conservation regions were restricted to exons of human and mouse lincRNAs (Fig. 3D).

### Expression of lincRNAs in different groups

**Figure 4:**
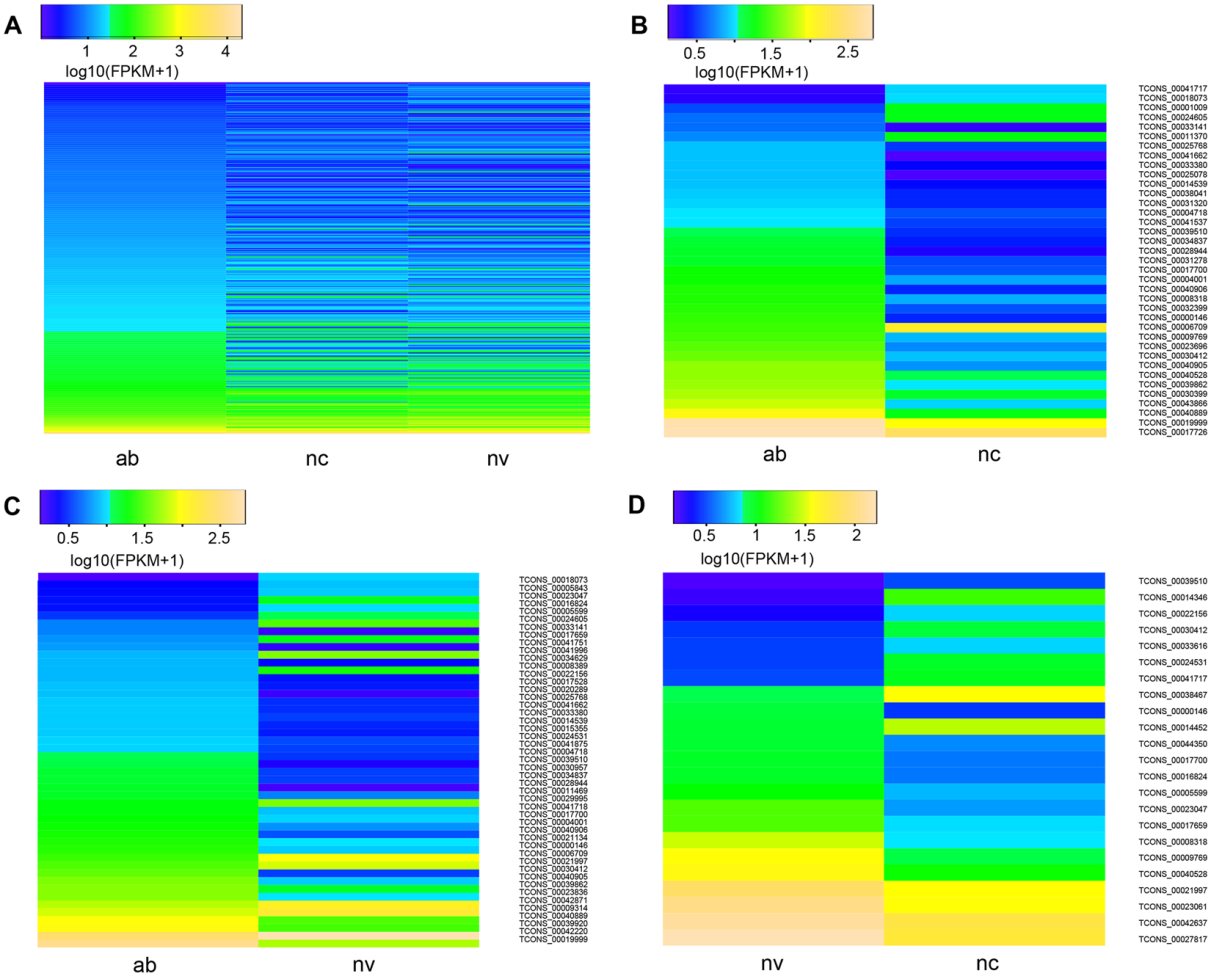
Expression of lincRNAs in different groups. Shown are heat maps of the log_10_ transformed FPKM + 1 expression values for differentially expressed lincRNAs. The density of the color scheme is calibrated to the log_10_ expression level, such that yellow refers to higher expression while blue refers to lower expression. The bar code represents the color scale of the log_10_(FPKM + 1). ab: the ab group; nc: the nc group; nv: the nv group. (**A**) All lincRNAs expression in three groups; (**B**) 37 differentially expressed lincRNAs between the ab group and the nc group; (**C**) 48 differentially expressed lincRNAs between the ab group and the nv group; (**D**) 23 differentially expressed lincRNAs between the nv group and the nc group.

Previous studies have shown that lincRNAs are expressed at significantly lower levels compared with protein-coding genes^21, 22^. Based on the expression levels estimated by Cufflinks for three transcriptomes, we compared the expression of lincRNAs and protein-coding genes in three groups. Our results showed that protein-coding genes have higher average expression level than that of lincRNAs in all three groups (protein_coding genes vs lincRNAs: 373.7 FPKM vs 95.2 FPKM in the ab group; 569.3 FPKM vs 105.6 FPKM in the nc group; 518.5 FPKM vs 114.0 FPKM in the nv group). To explore the function of lincRNAs, after profiling 323 lincRNAs expression in all groups (Fig. 4A), we conducted the differential expression analysis among the three groups by using Cuffdiff and detected 71 differentially expressed lincRNAs (DELs) through multiple comparisons among three groups by expression rate change. In detail, a total of 31 and 14 up-regulated and 6 and 9 down-regulated DELs were noted in the ab group and the nv group compared with the nc group, respectively, and 32 up-regulated and 16 down-regulated DELs were noted in the ab group compared with the nv group (Fig. 4B–D, Tables S2–S4).

### Association analysis of methylation and expression of lincRNAs

**Figure 5:**
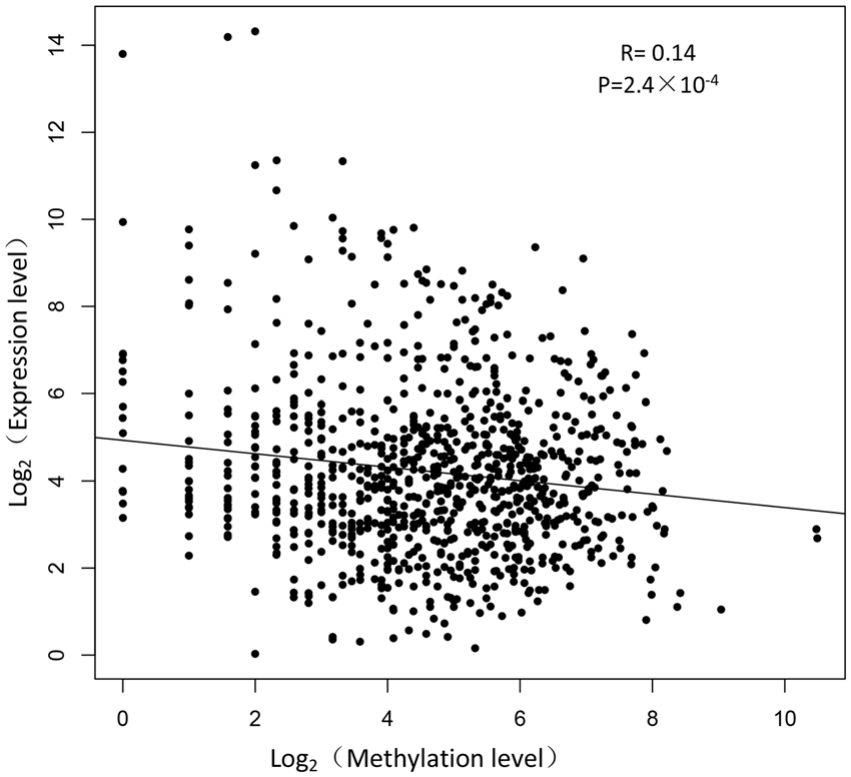
Scatter plot of the lincRNAs expression and methylation levels in three groups. The Pearson’s correlation was calculated between the log2 ratios of lincRNAs expression and the log2 ratios of lincRNAs methylation. The line represents regression line. The statistical significance was calculated by R language (version: 3.2.4).

Based on the data from methylated DNA immunoprecipitation sequencing (MeDIP-seq) in previous study^10^, we calculated the DNA methylation of lincRNA genes and explored the correlation between the methylation and expression of lincRNA genes. We found that the DNA methylation level in the promoter of lincRNA genes was significantly lower than that in the gene body region (P < 2.0e-4, Table S5), and this result was similar to protein-coding genes in previous studies^23, 24^. Numerous studies have proven that DNA methylation can regulate gene expression^25–27^, so we wondered whether any regulatory relationship exists between lincRNA gene methylation and their expression. Combining the lincRNAs methylation level and its expression level, we found that methylation in the promoter of lincRNA genes can significantly down-regulate its expression (P < 2.4e-5, Fig. 5), while no significant relationship was found between DNA methylation and expression in the gene body of lincRNA genes (P > 1.6e-1).

### Gene ontology and pathway analysis of PTGs of lincRNAs

**Figure 6:**
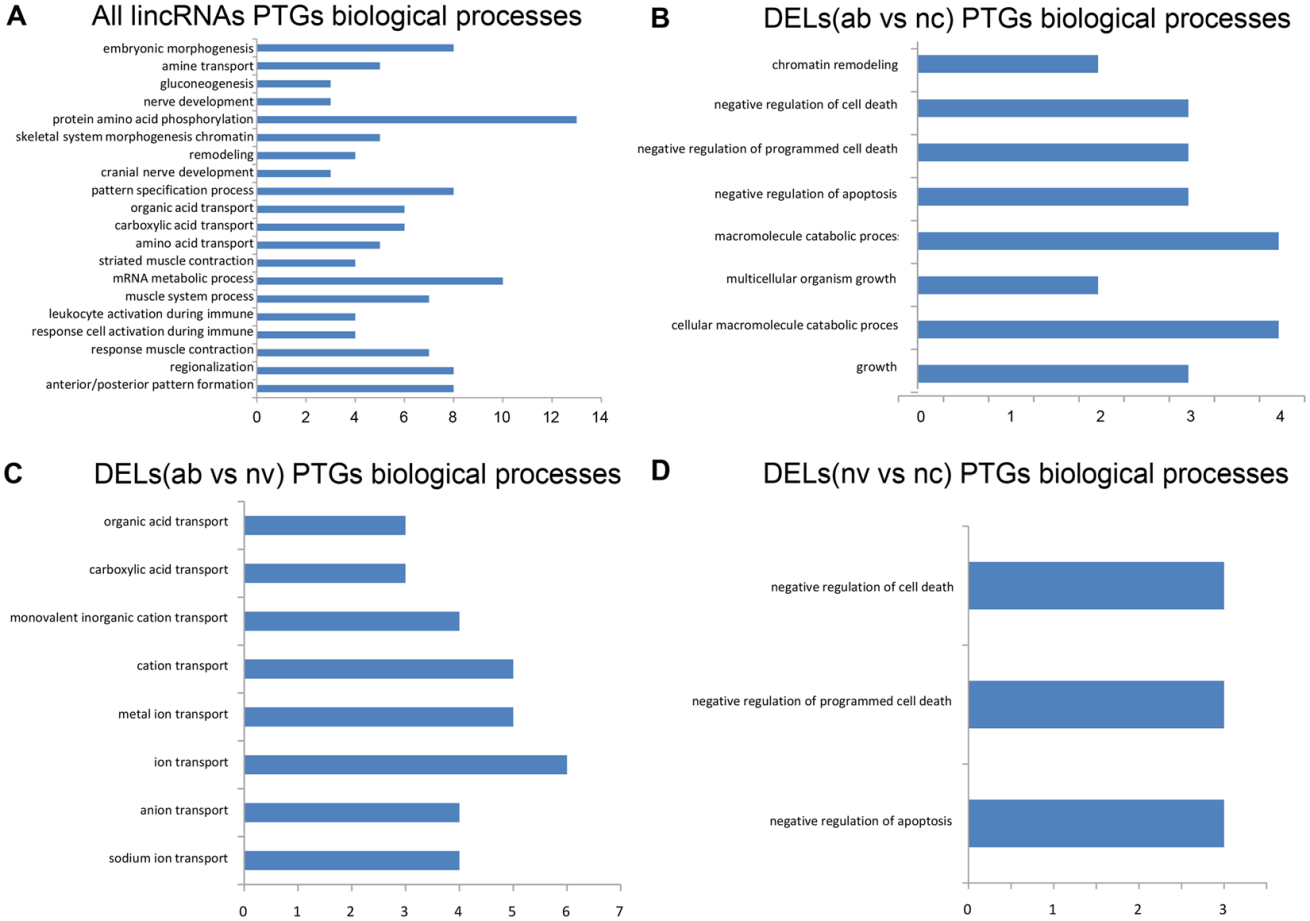
Gene ontology and pathway analysis of PTGs of lincRNAs. The x axis indicates the number of genes, and the y axis indicates different biological processes. ab: the ab group; nc: the nc group; nv: the nv group. (**A**) Biological processes of all lincRNAs PTGs; (**B**) Biological processes of PTGs of DELs (ab vs nc); (**C**) Biological processes of PTGs of DELs (ab vs nv); (**D**) Biological processes of PTGs of DELs (nv vs nc).

Most lincRNAs have not been functionally characterized because of its low expression, whereas many studies have proven that lncRNAs can cis-regulate protein-coding genes transcribed in close proximity to it through transcriptional interference^28–31^. Therefore those protein-coding genes that were transcribed nearby (<10 kb) lincRNAs may represent the best PTGs for interpreting the function of lincRNAs^7, 32^. We collected a total of 245 PTGs that were transcribed near their lincRNA loci, and of these 245 PTGs, 56 PTGs were transcribed near their DEL loci. Then DAVID analysis was performed by running queries for each PTG against the DAVID database. The results of the DAVID analysis showed that 57 of the 245 PTGs significantly participated in 32 biological processes (P < 0.05) (Fig. 6A, Table S6). In particular, some PTGs participated in muscle-related processes, such as muscle contraction, muscle system process, skeletal system morphogenesis and striated muscle contraction. In addition, 9 PTGs were significantly involved in the regulation of actin cytoskeleton, ErbB signalling pathway and Fc gamma R-mediated phagocytosis (P < 0.05). Meanwhile, we also performed DAVID analysis of 56 PTGs of the 71 DELs. The results of DAVID analysis showed that 8, 6 and 3 PTGs of DELs between two groups (the ab group vs the nc group, the ab group vs the nv group, the nv group vs the nc group) significantly participated in 8, 8 and 3 biological processes (P < 0.05) (Fig. 6B–D, Tables S7–S9), respectively, and most of these biological processes differed from those associated with the 245 protein-coding genes.

### Integrated analysis of promoter methylation and expression of lincRNAs and their PTGs expression

**Figure 7:**
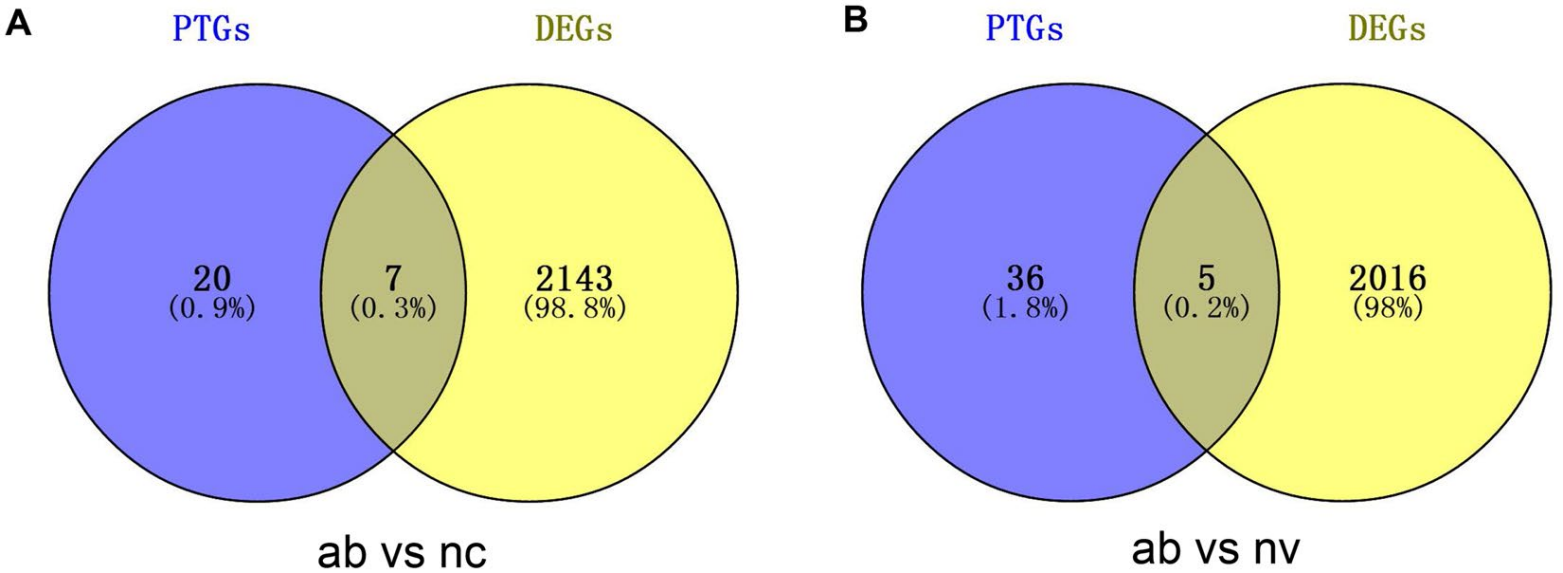
Numbers of DELs and DEGs in different groups. ab: the ab group; nc: the nc group; nv: the nv group. (**A**) Venny of DELs and DEGs between the ab group and the nc group; (**B**) Venny of DELs and DEGs between the ab group and the nv group.

Based on our previous researches, we counted the expression level of the 245 PTGs in three groups (Table S10)^9, 10^. Together with the expression level of lincRNAs, we found that no significant correlation exists between expressions of PTGs and their corresponding lincRNAs (P = 2.79e-1). Furthermore, we combined the PTGs with those differentially expressed genes (DEGs) in our previous studies to explore whether or not some intersections existed between them^9, 10^. Finally, we found a total of 50 PTGs of the lincRNAs that were differentially expressed among three groups through multiple comparisons. Of the aforementioned 50 PTGs, we found 11 PTGs whose corresponding lincRNAs were also differentially expressed among three groups (the ab group vs the nc group and the ab group vs the nv group) (Fig. 7A and B). With regard to the 11 PTGs, we found that almost all of them were up-regulated in the ab group compared with the nc and nv group (Table 2). Combining the promoter methylation and expression status of lincRNAs, we found that 7 of 11 lincRNAs exhibited the same regulatory relationship in which the lower promoter methylation of lincRNAs can up-regulate its expression and further up-regulate its PTGs expression, whereas 2 lincRNAs (TCONS_00025078 and TCONS_00034837) exhibited another different regulatory mechanism in which their high promoter methylation can up-regulate its expression and then up-regulate its PTGs expression. These results implied that multiple regulating relationships exist between lincRNAs and PTGs, and lincRNAs have unknown complicated regulation mechanisms in the abnormal phenotype of the cloned pigs.

**Table 2:** Promoter methylation and expression status of lincRNAs and their PTGs expression in different comparisons.

	lincRNAs	Promoter methylation status	Expression status	PTGs of lincRNA	Expression status of PTG
**ab vs nc**	TCONS_00043866	down^a^	up^b^	ENSSSCG00000028148(DMD)	down
	TCONS_00031320	down	up	ENSSSCG00000003559(ARID1A)	up
	TCONS_00038041	down	up	ENSSSCG00000014907(CREBZF)	up
	TCONS_00025078	up	up	ENSSSCG00000006490(SMG5)	up
	TCONS_00033141	down	up	ENSSSCG00000001959(CFL2)	up
	TCONS_00034837	up	up	ENSSSCG00000002539	up
	TCONS_00004001	down	up	ENSSSCG00000010860(PSEN2)	up
**ab vs nv**	TCONS_00021134	down	up	ENSSSCG00000014266(CDC42SE2)	up
	TCONS_00034629	down	up	ENSSSCG00000002281(CHURC1-FNTB)	up
	TCONS_00015355	down	up	ENSSSCG00000016808(C5orf22)	up
	TCONS_00004001	down	up	ENSSSCG00000010860(PSEN2)	up
	TCONS_00017659	down	down	ENSSSCG00000016646(IFRD1)	up

ab: the ab group; nc: the nc group; nv: the nv group. ^a^Indicates this lincRNA had lower expression level in the ab group compared to another group. ^b^Indicates this lincRNA had higher expression level in the ab group compared to another group.

### RNA-Seq data validation through qRT-PCR

**Figure 8:**
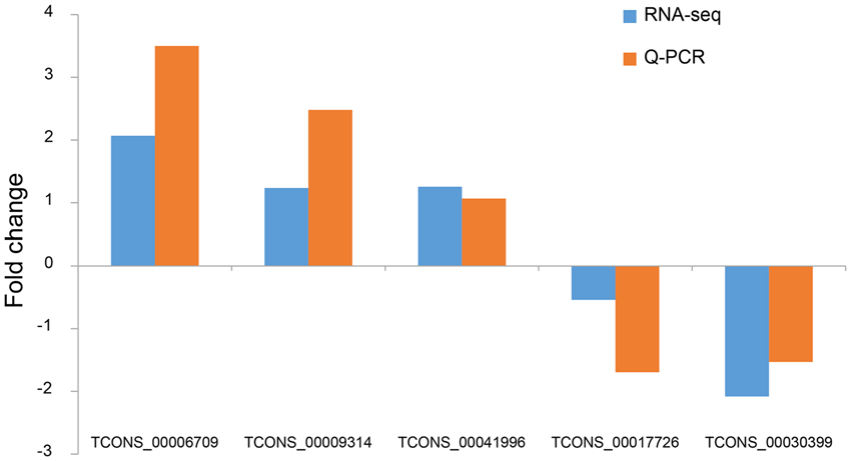
Validation of lincRNAs by qRT-PCR. The y-axis indicates the fold change of RNA-Seq and qRT-PCR.

Based on the RNA-Seq results, we randomly selected five lincRNAs (TCONS_00006709, TCONS_00009314, TCONS_00041996, TCONS_00017726 and TCONS_00030399) for qRT-PCR. We found that the expression status of five lincRNAs in different groups corresponded with that in RNA-seq results (Fig. 8), proving the reliability of our sequencing results.

## Discussion

In this study, we performed the identification and characterization of lincRNAs based on RNA-seq data in previous studies of our laboratory group^9, 10^. The size of pig genome is approximately the same as that of human and mouse; however, fewer lincRNAs identified in pig compare with the aforementioned two species^2, 18, 21, 33^, indicating that a large amount of pig lincRNAs are yet to be discovered. We identified a total of 152 novel lincRNAs, broadening the pig lincRNAs annotation. In our identification diagram, we combined CPC with Pfam search to reduce false negative and false positive results according to previous study^5^. Our putative lincRNAs displayed a series of characteristics, such as shorter transcripts, fewer exons, longer exon length and lower expression level in comparison with protein-coding genes, and this result corresponds with those of other studies^32, 34–36^. In our study, only a few lincRNAs had significant interrelation with human and mouse lincRNAs, and this result was in accordance with previous findings^6^. These conclusions indicated that our identification results were reliable. Our diagram can also help in the identification of lincRNAs in other species.

As a kind of skeletal muscle, the leg muscle can directly affect pig characteristics, for example, muscularity, through many physiological and metabolic processes^37, 38^. Previous studies on factors affecting the growth and development of skeletal muscle have mainly focused on protein-coding genes^39^, hormones^40^ and microRNA^41^ instead of lincRNAs. In this study, we identified 323 lincRNAs in the transcriptome of pig leg muscle. Some studies have reported that lincRNAs exhibited more tissue specificity than protein-coding genes^22, 42^; therefore, these lincRNAs may specifically express in leg muscle and participate in a series of muscle-related processes.

Numerous studies have reported that DNA methylation plays essential roles in embryo development and cell differentiation^43, 44^. However, these studies mainly focused on protein-coding genes, and only a few studies have reported about methylation of lincRNA genes. In previous study, Zhou *et al*. has characterized the DNA methylation pattern of pig lincRNA genes in adipose and muscle tissues. While, the relationship between the promoter methylation and expression of lincRNA genes has not been reported. In this study, we found a slightly negative correlation between the methylation of lincRNA gene promoter and lincRNA expression. In a previous study, Zhang *et al*. reported that lincRNA genes have higher methylation levels than that of protein-coding genes^45^; thus we inferred that increased methylation levels in lincRNA gene promoter may contribute to their lower expression levels compared with protein-coding genes.

Previous studies have demonstrated that lncRNAs have significant impact on gene regulation in cis^30, 46, 47^. In our study, we found a total of 245 protein-coding genes that were transcribed nearby 323 lincRNAs, indicating that majority of pig lincRNAs were transcribed nearby (<10 kb) protein-coding genes, and this result was also consistent with Bertone’s and Yu’s conclusion that lincRNAs were preferentially found within 10 kb of protein-coding genes^32, 48^. We explored the lincRNA function through gene ontology and pathway analysis of their PTGs. We found that some PTGs were involved in the regulation of muscle formation and contraction, which are inextricably related with the leg physiology. Considering the leg weakness of abnormal cloned piglets, we inferred that these PTGs may regulate the function of skeletal muscle through certain mechanisms that deserve further functional studies. In addition, we also identified some DELs between three groups, and the PTGs of these DELs were significantly involved in cell death and growth and ion transport-related processes; these functions were significantly related with skeletal muscle growth and development. Thus we concluded that the DELs between two groups may impact skeletal muscle performance by regulating their PTGs. The mechanism by which these DELs exert their regulatory function on PTGs and then affect the performance of the skeletal muscle is worth further research.

Moreover, we found that 50 PTGs of lincRNAs were also differentially expressed in our previous studies^9, 10^, giving us evidence that lincRNAs can regulate its PTGs to exert functions on skeletal muscle growth and development. Interestingly, by comparing two groups, we found 11 PTGs of DELs differentially expressed (Table 2). From multiple comparisons of three groups, we also found that the expression level of most PTGs of DELs was consistent with that of DEGs, and this result may mean that these DELs could positively regulate the expression of their PTGs. Considering the insignificant correlation between the expression level of lincRNAs and their PTGs, we can not draw a universal conclusion that lincRNAs positively or negatively regulate their PTGs expression. In the previous study, Wang *et al*. categorized lncRNAs into four categories, namely, signal, decoy, guide, and scaffold; and they reported that individual lncRNAs may possess one or several of these categories and exert different regulatory function^49, 50^. Thus, we hypothesized that some specific relationship may exist between lincRNAs and their target genes when these lincRNAs can be classified under the same category. Moreover, we noted that 7 of the 11 DELs whose PTGs were also differentially expressed in the ab group compared with the other 2 groups, had the same regulatory relationship in terms of promoter methylation and expression of lincRNAs and PTG expression, as described in the result section. Meanwhile, 4 remaining DELs exhibited three other regulatory mechanisms. Based on this result, we inferred that lincRNAs have a complicated mechanism in regulating muscle growth and development of pig at early stage; however, a majority of lincRNAs related to muscle growth and development can exert their functions through the regulatory mechanism, just like the 7 aforementioned DELs, and lead to muscle-related disabilities in cloned pigs.

In our study, we found that lincRNAs TCONS_00043866 was down-methylated and up-regulated, whereas the pig PTG Duchenne muscular dystrophy (DMD) gene was down-regulated in the ab group compared with the nc group. Previous studies have demonstrated that insufficient DMD abundance would lead to muscle weakness and dystrophy^51, 52^. Considering that the standing and walking disability of the abnormal cloned piglets was a typical muscle weakness, we inferred that low promoter methylation level of TCONS_00043866 in the ab group may up-regulate its expression which then negatively regulate the expression of DMD and contribute to the muscle weakness. Both the lincRNAs TCONS_00038041 and its PTG CREB/ATF BZIP transcription factor (CREBZF) were up-regulated in the ab group compared with the nc group, and TCONS_00038041 had a lower promoter methylation level in the ab group compared with the nc group. The bone morphogenetic proteins (BMPs) are essential for mesoderm formation, skeletal and limb development, and cellular differentiation^53–55^. Lee discovered that CREBZF can inhibit the function of BMPs by interacting with drosophila mothers against decapentaplegic protein (Smads)^56^. Meanwhile, Bodnarchuk and Zhang have demonstrated that CREBZF expression can supress cell growth and induce apoptosis^57, 58^. Therefore, we speculated that down-methylation of TCONS_00038041 may up-regulate its expression, then increase CREBZF expression, and finally lead to skeletal muscle cell death and unusual bone formation in the abnormal cloned piglets. A lincRNA TCONS_00004001 was up-regulated in the ab group compared with the nc and nv group, and its PTG presenilin protein2 (PSEN2) was also up-regulated in a two group comparison. Furthermore, TCONS_00004001 also had lower promoter methylation level in the ab group compared with the other two groups. Several studies have demonstrated that PSEN2 was a key regulator of Alzheimer’s disease by regulating neural development, and its up-expression can induce and/or proliferate a pro-inflammatory response in the brain of persons with Alzheimer disease^59, 60^. Muscle movement is regulated by the nervous system^61–63^; therefore, we inferred that down-methylation of TCONS_00004001 can up-regulate its expression in the ab group and then affect the skeletal muscle function through the nervous system by enhancing PSEN2 expression in the ab group.

In conclusion, we identified a number of reliable novel lincRNAs in porcine leg muscle and found the correlation between lincRNA gene methylation and its expression. We presented a new regulatory mechanism in which lincRNA methylation may affect its expression, then affect its PTGs expression, and finally impact muscle performance. Although we listed a few typical lincRNAs that may contribute to the muscle weakness, the mechanism by which these lincRNAs exert function through their PTGs is unknown. Moreover, functions of several lincRNAs are still unclear; thus, further functional studies are needed. These lincRNAs, particularly DELs with PTGs differentially expressed in three groups, represent ideal candidates for further studies about those genes in the processes of skeletal muscle growth and development.

## Materials and Methods

### Ethics statement

In this study, the sows used for RNA-seq and MeDIP-seq were raised in the Animal Science Research Center with standard rations and water at the University of Missouri. Animal care and all experimentation were conducted in accordance with the guidelines pre-approved by the University of Missouri Institutional Animal Care and Use Committee (#s 3319 and 3947). All samples were taken from the same part of the leg as described by Li *et al*.^10^.

### Datasets

All RNA-seq and MeDIP-seq data of piglets leg muscle used in this study were from previous studies in our laboratory group^9, 10^, which were uploaded to the NCBI Gene Expression Omnibus (GEO) databases with accession number listed in Table 1. Both RNA-seq data and MeDIP data included three groups (the abnormal cloned group (ab), the normal cloned group (nc) and the normal *in vivo* group (nv; conventionally bred group) totalling 45 million and 147 million reads generated by IlluminaHiSeq.2000, respectively. The Ensembl^64^ gene annotations of pig which we used were downloaded from ftp://ftp.ensembl.org/pub/release-75/gtf/sus_scrofa/. The pig lincRNAs reference and annotations were downloaded from http://res.xaut.edu.cn/aldb/download.jsp ^65^, the RefSeq NR database was downloaded from ftp://ftp.ncbi.nih.gov/blast/db/, and the human and mouse lincRNAs references were downloaded from http://asia.ensembl.org/info/data/ftp/index.html.

### Initial assembly

The raw RNA-seq reads were first mapped to the pig reference genome (*Sus scrofa* 10.2, http://ftp.ensembl.org/pub/release-75/fasta/sus_scrofa/dna/) by Tophat 2.0.13 with default parameters^66^. Meanwhile, we set the “−G” option of Tophat together with the Gene Transfer Format (GTF) file of Ensembl gene annotation for reads mapping. The mapped reads were assembled through Cufflinks 2.1.1 with default parameters^67^. Cufflinks uses a probabilistic model to assemble and quantify the expression level of a minimal set of isoforms and provides a maximum likelihood explanation of the expression data in given loci. Three assembled transcript files (GTF format) of three groups were then merged into a unique transcriptome using Cuffmerge utility provided by the Cufflinks package. The lincRNAs detection pipeline was used to filter the merged assembly.

### Pipeline for the identification of lincRNAs

We used following steps to identify lincRNAs from the pig leg muscle transcriptome: (1) only transcripts with ‘u’ category categorized by Cuffmerge which indicated intergenic transcripts were retained; (2) transcripts with single exon or less than 200 bp in length were removed; (3) the Coding Potential Calculator (CPC) tool^20^ was used to assess the coding potential of transcripts in both strands, and only transcripts with CPC value <0 in both strands were retained; (4) transcripts that contained known protein domain were filtered. To accomplish this, we translated transcripts sequence into six possible protein sequence by Transeq (http://www.ebi.ac.uk/Tools/st/emboss_transeq/), and then transcripts with any possible protein sequence significantly (E-value < 1e-5) hit in the Pfam (http://pfam.xfam.org/search) database were filtered; (5) to minimize false positive, we selected transcripts that had detectable expression in all three groups.

### Comparisons between lincRNAs and protein-coding transcripts

We firstly selected transcripts with ‘=’ category which means completely matched with reference genome from the merged assembly and then transcripts annotated as “protein-coding” in pig Ensembl gene annotation were retained, finally we got a total of 31,744 protein-coding transcripts. We then compared the lincRNAs with these protein-coding transcripts in terms of transcript length, exon number and length, and expression level. We used R language 3.2.4 to judge the differences between lincRNAs and protein-coding transcripts, and less than 0.05 was considered significant.

### Differential expression lincRNAs analysis

We used Cuffdiff utility provided by the Cufflinks package to conduct differential expression tests on multiple comparisons among three groups. The fold changes were calculated via log_2_(FPKM1/FPKM2) (FPKM: Fragments per kilobase of transcript per million mapped reads). A transcript will be identified differentially expressed in two groups if the fold change was bigger than 1 and the FDR-adjusted p-value after Benjamini-Hochberg correction namely q-value given in the test less than 0.05^68^.

### Analysis of DNA methylation of lincRNA genes

The raw MeDIP-seq reads were first mapped to the pig reference genome (*Sus scrofa* 10.2, http://ftp.ensembl.org/pub/release-75/fasta/sus_scrofa/dna/) by Bowtie2 2.2.3^69^ with default parameters. Then HTSeq-count^70^ was used to calculated the methylation level of each lincRNA genes, and we used normalized reads number to represent the methylation level of each lincRNA genes. We defined the promoter region as the upstream 2 kb of the transcription start site of lincRNA genes.

### Predication and DAVID analysis of PTGs of lincRNAs

Based on the assembly result, we had the position information of each transcript. We defined a lincRNA PTG as protein-coding genes that were transcribed nearby (<10 kb) lincRNAs, and we got all PTGs by BEDTools 2.17.0^71^. Then we performed DAVID (Database for Annotation, Visualization and Integrated Discovery) analysis by running queries for each PTG against the DAVID database^72^. Because of the limited annotation of the porcine genome, all PTGs were firstly converted into human homologous genes using BIOMART from Ensembl (http://www.ensembl.org/biomart/martview/8143dcf2a64771c957b28d28832759b6).

### Quantification of lincRNAs through quantitative reverse transcription polymerase chain reaction (qRT-PCR)

RNA samples were available in our laboratory and had been described in our previous studies^9, 10^. We confirmed five lincRNAs in three pooled RNA libraries, and each lincRNA had three technical repeats. qRT-PCR was performed with SYBR Green (Bio-Rad) to validate the RNA-Seq results. Five pairs of primers for qRT-PCR were designed using the Oligo 7 program (Table S11). The 18 s rRNA served as the endogenous control gene. The qRT-PCR data were analysed using the method described in previous study^73^. We used fold change to judge whether qRT-PCR results were in accordance with the RNA-Seq results.

## Electronic supplementary material

Supplementary Tables

## References

[1] Harrow J (2012). GENCODE: the reference human genome annotation for The ENCODE Project. Genome Res.

[2] Guttman M (2009). Chromatin signature reveals over a thousand highly conserved large non-coding RNAs in mammals. Nature.

[3] Huttenhofer A, Schattner P, Polacek N (2005). Non-coding RNAs: hope or hype?. Trends Genet.

[4] Loewer S (2010). Large intergenic non-coding RNA-RoR modulates reprogramming of human induced pluripotent stem cells. Nat Genet.

[5] Pauli A (2012). Systematic identification of long noncoding RNAs expressed during zebrafish embryogenesis. Genome Res.

[6] Ulitsky I, Shkumatava A, Jan CH, Sive H, Bartel DP (2011). Conserved function of lincRNAs in vertebrate embryonic development despite rapid sequence evolution. Cell.

[7] Zhao W (2015). Systematic identification and characterization of long intergenic non-coding RNAs in fetal porcine skeletal muscle development. Sci Rep.

[8] Yang Y (2017). Comparative analysis of DNA methylome and transcriptome of skeletal muscle in lean-, obese-, and mini-type pigs. Sci Rep.

[9] Zou C (2016). Genome-wide gene expression and DNA methylation differences in abnormally cloned and normally natural mating piglets. Anim Genet.

[10] Li G (2014). Dysregulation of genome-wide gene expression and DNA methylation in abnormal cloned piglets. BMC Genomics.

[11] Billerey C (2014). Identification of large intergenic non-coding RNAs in bovine muscle using next-generation transcriptomic sequencing. BMC Genomics.

[12] Lv J (2015). Identification of 4438 novel lincRNAs involved in mouse pre-implantation embryonic development. Mol Genet Genomics.

[13] Zhou ZY (2014). Genome-wide identification of long intergenic noncoding RNA genes and their potential association with domestication in pigs. Genome Biol Evol.

[14] Wu G (2014). LincRNA-p21 regulates neointima formation, vascular smooth muscle cell proliferation, apoptosis, and atherosclerosis by enhancing p53 activity. Circulation.

[15] Zhou L (2015). Linc-YY1 promotes myogenic differentiation and muscle regeneration through an interaction with the transcription factor YY1. Nat Commun.

[16] Sakakibara I, Santolini M, Ferry A, Hakim V, Maire P (2014). Six homeoproteins and a Iinc-RNA at the fast MYH locus lock fast myofiber terminal phenotype. PLoS Genet.

[17] Derrien T (2012). The GENCODE v7 catalog of human long noncoding RNAs: analysis of their gene structure, evolution, and expression. Genome Res.

[18] Guttman M (2010). Ab initio reconstruction of cell type-specific transcriptomes in mouse reveals the conserved multi-exonic structure of lincRNAs. Nat Biotechnol.

[19] Trapnell C (2012). Differential gene and transcript expression analysis of RNA-seq experiments with TopHat and Cufflinks. Nat Protoc.

[20] Kong L (2007). CPC: assess the protein-coding potential of transcripts using sequence features and support vector machine. Nucleic Acids Res.

[21] Cabili MN (2011). Integrative annotation of human large intergenic noncoding RNAs reveals global properties and specific subclasses. Genes Dev.

[22] Luo H (2013). Comprehensive characterization of 10,571 mouse large intergenic noncoding RNAs from whole transcriptome sequencing. PLoS One.

[23] Huang YZ (2014). Genome-wide DNA methylation profiles and their relationships with mRNA and the microRNA transcriptome in bovine muscle tissue (Bos taurine). Sci Rep.

[24] Lee JR (2014). Genome-wide analysis of DNA methylation patterns in horse. BMC Genomics.

[25] Shen L (2016). Genome-wide landscape of DNA methylomes and their relationship with mRNA and miRNA transcriptomes in oxidative and glycolytic skeletal muscles. Sci Rep.

[26] Schachtschneider KM (2015). Adult porcine genome-wide DNA methylation patterns support pigs as a biomedical model. BMC Genomics.

[27] Peat JR, Reik W (2012). Incomplete methylation reprogramming in SCNT embryos. Nat Genet.

[28] Jia H (2010). Genome-wide computational identification and manual annotation of human long noncoding RNA genes. RNA.

[29] Ponting CP, Oliver PL, Reik W (2009). Evolution and functions of long noncoding RNAs. Cell.

[30] Orom UA (2010). Long noncoding RNAs with enhancer-like function in human cells. Cell.

[31] Wang KC (2011). A long noncoding RNA maintains active chromatin to coordinate homeotic gene expression. Nature.

[32] Yu H, Zhao X, Li Q (2016). Genome-wide identification and characterization of long intergenic noncoding RNAs and their potential association with larval development in the Pacific oyster. Sci Rep.

[33] Managadze D (2013). The vast, conserved mammalian lincRNome. PLoS Comput Biol.

[34] Wu Y (2016). Systematic Identification and Characterization of Long Non-Coding RNAs in the Silkworm, Bombyx mori. PLoS One.

[35] Wang J (2016). Identification and Functional Prediction of Large Intergenic Noncoding RNAs (lincRNAs) in Rainbow Trout (Oncorhynchus mykiss). Mar Biotechnol (NY).

[36] Zhang YC (2014). Genome-wide screening and functional analysis identify a large number of long noncoding RNAs involved in the sexual reproduction of rice. Genome Biol.

[37] Egan B, Zierath JR (2013). Exercise metabolism and the molecular regulation of skeletal muscle adaptation. Cell Metab.

[38] Nitert MD (2012). Impact of an exercise intervention on DNA methylation in skeletal muscle from first-degree relatives of patients with type 2 diabetes. Diabetes.

[39] Olson EN (1991). Molecular control of myogenesis: antagonism between growth and differentiation. Mol Cell Biochem.

[40] Gaster M, Staehr P, Beck-Nielsen H, Schroder HD, Handberg A (2001). GLUT4 is reduced in slow muscle fibers of type 2 diabetic patients: is insulin resistance in type 2 diabetes a slow, type 1 fiber disease?. Diabetes.

[41] Zhang D (2014). Thyroid hormone regulates muscle fiber type conversion via miR-133a1. J Cell Biol.

[42] Lv J (2014). Identification and characterization of long intergenic non-coding RNAs related to mouse liver development. Mol Genet Genomics.

[43] Meissner A (2010). Epigenetic modifications in pluripotent and differentiated cells. Nat Biotechnol.

[44] Reik W (2007). Stability and flexibility of epigenetic gene regulation in mammalian development. Nature.

[45] Zhou ZY (2015). DNA methylation signatures of long intergenic noncoding RNAs in porcine adipose and muscle tissues. Sci Rep.

[46] Nagano T (2008). The Air noncoding RNA epigenetically silences transcription by targeting G9a to chromatin. Science.

[47] Kim TK (2010). Widespread transcription at neuronal activity-regulated enhancers. Nature.

[48] Bertone P (2004). Global identification of human transcribed sequences with genome tiling arrays. Science.

[49] Wang KC, Chang HY (2011). Molecular mechanisms of long noncoding RNAs. Mol Cell.

[50] Da Sacco L, Baldassarre A, Masotti A (2012). Bioinformatics tools and novel challenges in long non-coding RNAs (lncRNAs) functional analysis. Int J Mol Sci.

[51] Hollinger K (2014). Dystrophin insufficiency causes selective muscle histopathology and loss of dystrophin-glycoprotein complex assembly in pig skeletal muscle. FASEB J.

[52] Klymiuk N (2013). Dystrophin-deficient pigs provide new insights into the hierarchy of physiological derangements of dystrophic muscle. Hum Mol Genet.

[53] Wozney JM (1988). Novel regulators of bone formation: molecular clones and activities. Science.

[54] Hogan BL (1996). Bone morphogenetic proteins: multifunctional regulators of vertebrate development. Genes Dev.

[55] Zhao GQ (2003). Consequences of knocking out BMP signaling in the mouse. Genesis.

[56] Lee JH (2012). CREBZF, a novel Smad8-binding protein. Mol Cell Biochem.

[57] Bodnarchuk TW, Napper S, Rapin N, Misra V (2012). Mechanism for the induction of cell death in ONS-76 medulloblastoma cells by Zhangfei/CREB-ZF. J Neurooncol.

[58] Zhang R, Thamm DH, Misra V (2015). The effect of Zhangfei/CREBZF on cell growth, differentiation, apoptosis, migration, and the unfolded protein response in several canine osteosarcoma cell lines. BMC Vet Res.

[59] Riazanskaia N (2002). Regulatory region variability in the human presenilin-2 (PSEN2) gene: potential contribution to the gene activity and risk for AD. Mol Psychiatry.

[60] Sannerud R (2016). Restricted Location of PSEN2/gamma-Secretase Determines Substrate Specificity and Generates an Intracellular Abeta Pool. Cell.

[61] Weavil, J. C., Sidhu, S. K., Mangum, T. S., Richardson, R. S. & Amann, M. Fatigue diminishes motoneuronal excitability during cycling exercise. *J Neurophysiol* jn 00300 2016 (2016). 10.1152/jn.00300.2016 PMC5144707 27440242

[62] Deries, M. & Thorsteinsdottir, S. Axial and limb muscle development: dialogue with the neighbourhood. *Cell Mol Life Sci* (2016). 10.1007/s00018-016-2298-7 PMC11108464 27344602

[63] Dres, M. *et al*. Coexistence and Impact of Limb Muscle and Diaphragm Weakness at Time of Liberation From Mechanical Ventilation in Medical ICU Patients. *Am J Respir Crit Care Med* (2016). 10.1164/rccm.201602-0367OC 27310484

[64] Flicek P (2013). Ensembl 2013. Nucleic Acids Res.

[65] Sun L (2013). Utilizing sequence intrinsic composition to classify protein-coding and long non-coding transcripts. Nucleic Acids Res.

[66] Trapnell C, Pachter L, Salzberg SL (2009). TopHat: discovering splice junctions with RNA-Seq. Bioinformatics.

[67] Trapnell C (2010). Transcript assembly and quantification by RNA-Seq reveals unannotated transcripts and isoform switching during cell differentiation. Nat Biotechnol.

[68] Benjamini Y, Drai D, Elmer G, Kafkafi N, Golani I (2001). Controlling the false discovery rate in behavior genetics research. Behav Brain Res.

[69] Langmead B, Salzberg SL (2012). Fast gapped-read alignment with Bowtie 2. Nat Methods.

[70] Anders S, Pyl PT, Huber W (2015). HTSeq–a Python framework to work with high-throughput sequencing data. Bioinformatics.

[71] Quinlan AR, Hall IM (2010). BEDTools: a flexible suite of utilities for comparing genomic features. Bioinformatics.

[72] Huang da, W. *et al*. Extracting biological meaning from large gene lists with DAVID. *Curr Protoc Bioinformatics* Chapter 13, Unit 13 11 (2009). 10.1002/0471250953.bi1311s27 19728287

[73] Dalkilic I, Schienda J, Thompson TG, Kunkel LM (2006). Loss of FilaminC (FLNc) results in severe defects in myogenesis and myotube structure. Mol Cell Biol.

